# Genetic profile of syndromic retinitis pigmentosa in Portugal

**DOI:** 10.1007/s00417-023-06360-2

**Published:** 2024-01-08

**Authors:** Telmo Cortinhal, Cristina Santos, Sara Vaz-Pereira, Ana Marta, Lilianne Duarte, Vitor Miranda, José Costa, Ana Berta Sousa, Virginie G. Peter, Karolina Kaminska, Carlo Rivolta, Ana Luísa Carvalho, Jorge Saraiva, Célia Azevedo Soares, Rufino Silva, Joaquim Murta, Luísa Coutinho Santos, João Pedro Marques

**Affiliations:** 1 Department of Ophthalmology, Centro Hospitalar e Universitário de Coimbra (CHUC), Coimbra, Portugal; 2 Instituto de Oftalmologia Dr. Gama Pinto (IOGP), Lisboa, Portugal; 3 iNOVA4Health, NOVA Medical School, Faculdade de Ciências Médicas, NMS, FCM, Universidade NOVA de Lisboa, Lisboa, Portugal; 4 Department of Ophthalmology, Centro Hospitalar Universitário de Lisboa Norte (CHULN), Lisboa, Portugal; 5 Department of Ophthalmology, Faculdade de Medicina, Universidade de Lisboa, Lisbon, Portugal; 6 Department of Ophthalmology, Centro Hospitalar e Universitário de Santo António (CHUdSA), Porto, Portugal; 7 Instituto Ciências Biomédicas Abel Salazar (ICBAS), Porto, Portugal; 8 Department of Ophthalmology, Centro Hospitalar de Entre Douro e Vouga (CHEDV), Santa Maria da Feira, Portugal; 9 Department of Ophthalmology, Hospital de Braga (HB), Braga, Portugal; 10 Medical Genetics Unit, Hospital Pediátrico, Centro Hospitalar e Universitário de Lisboa Norte (CHULN), Lisboa, Portugal; 11 Institute of Molecular and Clinical Ophthalmology Basel (IOB), 4031 Basel, Switzerland; 12 Department of Ophthalmology, University of Basel, 4031 Basel, Switzerland; 13 Department of Ophthalmology, Inselspital, Bern University Hospital, 3010 Bern, Switzerland; 14 Department of Genetics and Genome Biology, University of Leicester, Leicester, LE1 7RH United Kingdom; 15 Medical Genetics Unit, Centro Hospitalar e Universitário de Coimbra (CHUC), Coimbra, Portugal; 16 Clinical Academic Center of Coimbra (CACC), Coimbra, Portugal; 17 University Clinic of Medical Genetics, Faculty of Medicine, University of Coimbra (FMUC), Coimbra, Portugal; 18 University Clinic of Pediatrics, Faculty of Medicine, University of Coimbra (FMUC), Coimbra, Portugal; 19 Medical Genetics Department, Centro de Genética Médica Jacinto Magalhães, Centro Hospitalar e Universitário do Porto (CHUP), Porto, Portugal; 20 Unit for Multidisciplinary Research in Biomedicine, Instituto de Ciências Biomédicas Abel Salazar, Universidade do Porto, Porto, Portugal; 21 Medical Science Department, Universidade de Aveiro, Aveiro, Portugal; 22 i3S - Instituto de Investigação e Inovação em Saúde, Universidade do Porto, Porto, Portugal; 23 University Clinic of Ophthalmology, Faculty of Medicine, University of Coimbra (FMUC), Coimbra, Portugal

**Keywords:** Inherited retinal diseases, Syndromic retinitis pigmentosa, Ophthalmic genetics, Genotype

## Abstract

**Purpose:**

Retinitis pigmentosa (RP) comprises a genetically and clinically heterogeneous group of inherited retinal degenerations, where 20–30% of patients exhibit extra-ocular manifestations (syndromic RP). Understanding the genetic profile of RP has important implications for disease prognosis and genetic counseling. This study aimed to characterize the genetic profile of syndromic RP in Portugal.

**Methods:**

Multicenter, retrospective cohort study. Six Portuguese healthcare providers identified patients with a clinical diagnosis of syndromic RP and available genetic testing results. All patients had been previously subjected to a detailed ophthalmologic examination and clinically oriented genetic testing. Genetic variants were classified according to the American College of Medical Genetics and Genomics; only likely pathogenic or pathogenic variants were considered relevant for disease etiology.

**Results:**

One hundred and twenty-two patients (53.3% males) from 100 families were included. Usher syndrome was the most frequent diagnosis (62.0%), followed by Bardet-Biedl (19.0%) and Senior-Løken syndromes (7.0%). Deleterious variants were identified in 86/100 families for a diagnostic yield of 86.0% (87.1% for Usher and 94.7% for Bardet-Biedl). A total of 81 genetic variants were identified in 25 different genes, 22 of which are novel. *USH2A* and *MYO7A* were responsible for most type II and type I Usher syndrome cases, respectively. *BBS1* variants were the cause of Bardet-Biedl syndrome in 52.6% of families. Best-corrected visual acuity (BCVA) records were available at baseline and last visit for 99 patients (198 eyes), with a median follow-up of 62.0 months. The mean BCVA was 56.5 ETDRS letters at baseline (Snellen equivalent ~ 20/80), declining to 44.9 ETDRS letters (Snellen equivalent ~ 20/125) at the last available follow-up (*p* < 0.001).

**Conclusion:**

This is the first multicenter study depicting the genetic profile of syndromic RP in Portugal, thus contributing toward a better understanding of this heterogeneous disease group. Usher and Bardet-Biedl syndromes were found to be the most common types of syndromic RP in this large Portuguese cohort. A high diagnostic yield was obtained, highlighting current genetic testing capabilities in providing a molecular diagnosis to most affected individuals. This has major implications in determining disease-related prognosis and providing targeted genetic counseling for syndromic RP patients in Portugal.

**Supplementary information:**

The online version contains supplementary material available at 10.1007/s00417-023-06360-2.


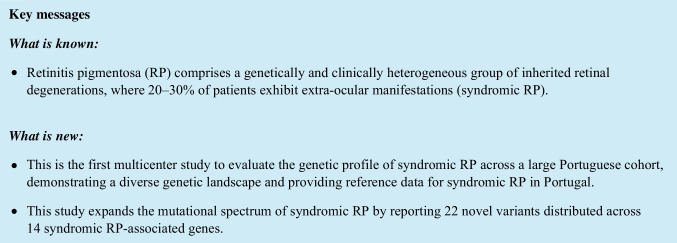


## Introduction

Retinitis pigmentosa (RP) comprises a genetically and clinically diverse group of inherited retinal degenerations (IRDs), primarily characterized by rod-cone degeneration. With an estimated prevalence of 1:4000 individuals, it is the most frequent form of IRD [1]. While most cases of RP are not associated with systemic abnormalities, 20–30% of patients exhibit extra-ocular disease and are referred to as syndromic RP [1–3]. Usher syndrome features sensorineural hearing loss (and in some forms vestibular impairment) in association with RP and is overall the most frequent form of syndromic RP [2–4], followed by Bardet-Biedl syndrome. In the latter, polydactyly, intellectual disability, and truncal obesity are among the most prevalent extra-ocular manifestations [2–4].

Genetic profiling of IRDs takes on an ever-growing significance for the affected individual, not only with regard to disease prognosis and genetic counseling but also for treatment prospects [5], which recently became a reality with the introduction of gene therapy for *RPE65*-associated retinal degeneration [6]. Even though therapies targeting the retinal phenotype of syndromic RP are not currently available, the genetic landscape of syndromic RP has been receiving increased interest worldwide, including a few European studies [7–10]. Although there are some similarities in genetic profiles, there is significant variation among regions and ethnic groups. This genetic diversity between populations may be partly explained by founder mutations [8, 11, 12], thus highlighting the importance of obtaining reference population-based data.

In Portugal, data on the genetic architecture of syndromic RP is currently scarce. By conducting a national, multicenter study, we aimed at characterizing the genetic landscape of syndromic RP in a large Portuguese cohort.

## Methods

### Study design

A nationwide, multicenter, retrospective cohort study was conducted in six Portuguese public healthcare providers (HCP): *Centro Hospitalar e Universitário de Coimbra* (CHUC), *Instituto de Oftalmologia Dr. Gama Pinto* (IOGP), *Centro Hospitalar Universitário de Lisboa Norte* (CHULN), *Centro Hospitalar e Universitário de Santo António* (CHUdSA), *Centro Hospitalar de Entre o Douro e Vouga* (CHEDV), and *Hospital de Braga* (HB). Patients with a clinical diagnosis of syndromic RP and available genetic testing results were retrieved from internal databases and the IRD-PT registry [12]. Every patient provided written informed consent prior to enrollment, and the study complied with the tenets of the Declaration of Helsinki for biomedical research. Of note, even though most of the data shown here has never been published, the study includes data that has been featured in previous publications [13–15].

### Clinical/demographic features

Data regarding demographics (age, gender, district of residence), family history, presence of consanguinity, age of ophthalmologic symptom onset, presence of ocular and systemic comorbidities, best-corrected visual acuity (BCVA) at baseline, and last available follow-up was obtained from each patient clinical record. A clinical diagnosis was established based on history and compatible structural (multimodal retinal imaging) and functional (electrophysiology testing and visual field testing) retinal findings. However, such testing was not standardized among the different contributing HCPs.

### Genetic testing

Peripheral blood samples were collected, and genomic DNA was isolated using a DNA extraction and purification kit based on the manufacturer’s protocol. A clinically oriented next-generation sequencing (NGS) approach was used, comprising whole-exome sequencing (WES) or WES-based NGS panels with copy number variation (CNV) screening, complemented by multiplex ligation-dependent probe amplification (MLPA), when necessary. Whenever possible, segregation analysis was performed on family members. Identified genetic variants were classified in compliance with the American College of Medical Genetics and Genomics (ACMG) standards and guidelines for the interpretation of sequence variants [16]. Only class IV (likely pathogenic) and class V (pathogenic) variants were deemed relevant to disease etiology. Variants were considered novel in the absence of previous reports featured in scientific publications. Genetic counseling provided by a medical geneticist was granted to all families.

### Statistical analysis

Statistical analysis was performed using the software IBM SPSS Statistics version 26 (Armonk, New York, USA). Descriptive statistics were computed for all variables. A statistically significant result was defined as a *p*-value < 0.05.

## Results

### Clinical/demographic features

**Fig. 1:**
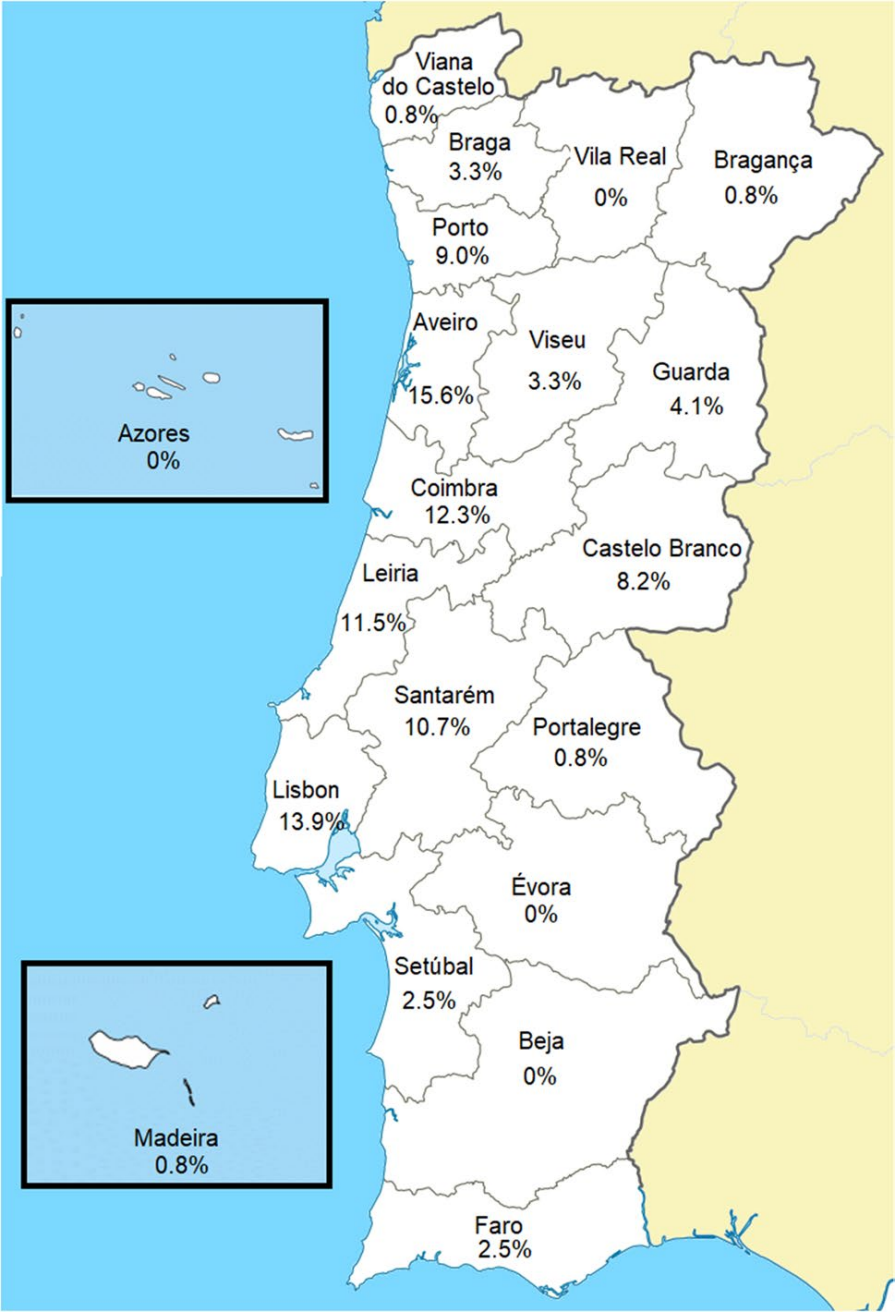
Cohort distribution by district of residence (data presented *per* patient)

A total of 122 patients (100 different families) with a clinical diagnosis of syndromic RP and available genetic testing results were included (75 patients from CHUC, 26 from IOGP, 7 from CHULN, 7 from CHUdSA, 5 from CHEDV and 2 from HB). Most patients (53.3%) were males, and the mean age was 44.6 ± 15.1 years (range 11–79). Family history of the disease was present in 53.3%, while 36.1% of patients reported consanguinity. Age of ophthalmic disease onset, defined as the first instance of RP-attributable symptoms, along with the demographic characterization of the cohort, is presented in Table 1, while the cohort distribution *per* district of residence is presented in Fig. 1.

**Table 1:** Demographic characterization of the cohort

Number of families (number of patients)		100 (122)		
Male gender: *n* (%)		65 (53.3%)		
Age: mean ± SD (years)		44.6 ± 15.1		
Family history, *n* (%)		65 (53.3%)		
Consanguinity, *n* (%)		44 (36.1%)		
Age of symptom onset, *n* (%)	Diagnosis	All patients	Usher syndrome	BBS
	≤ 5 years	19 (15.6%)	10 (13.5%)	6 (24.0%)
	6–10 years	27 (22.1%)	12 (16.2%)	10 (40.0%)
	11–20 years	29 (23.8%)	20 (27.0%)	2 (8.0%)
	21–30 years	14 (11.5%)	8 (10.8%)	1 (4.0%)
	31–50 years	15 (12.3%)	14 (18.9%)	0 (0%)
	Unknown	18 (14.8%)	10 (13.5%)	6 (24%)

Data presented *per* patient. Age of symptom onset is presented for all patients and the two most common diagnoses

*BBS*, Bardet-Biedl syndrome

**Fig. 2:**
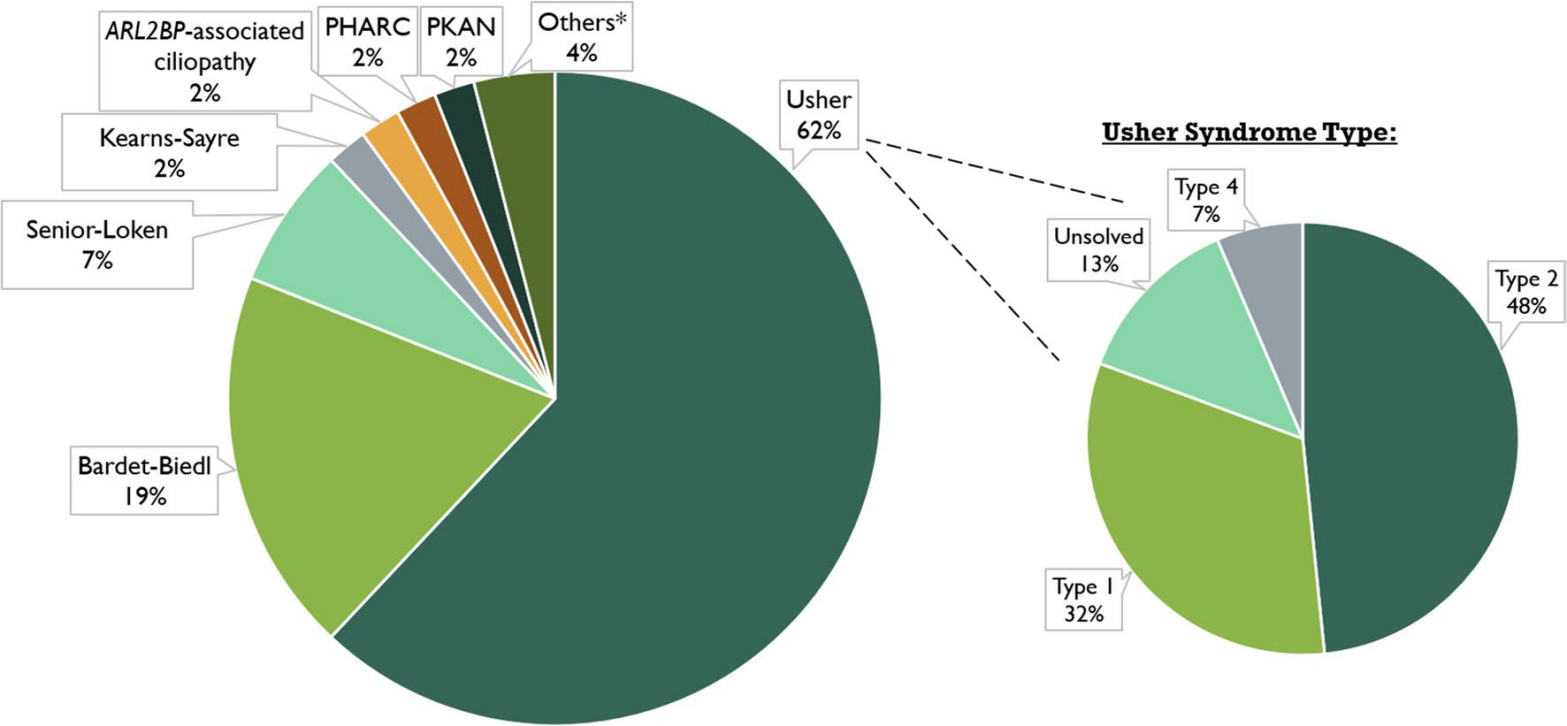
Cohort diagnosis distribution (percentage *per* family). *Others include bone marrow failure syndrome type 3; neuropathy, ataxia, retinitis pigmentosa (NARP) syndrome; Jalili syndrome; and mitochondrial DNA depletion syndrome. PHARC: polyneuropathy, hearing loss, ataxia, retinitis pigmentosa, and cataract; PKAN: pantothenate kinase-associated neurodegeneration

The most frequently encountered diagnosis was Usher syndrome, present in 62.0% of the families, followed by Bardet-Biedl (19.0%) and Senior-Løken (7.0%) syndromes. The remaining cases consisted of Kearns-Sayre syndrome (*n* = 2); *ARL2BP*-associated ciliopathy [14] (*n* = 2); polyneuropathy, hearing loss, ataxia, retinitis pigmentosa, and cataract (PHARC) (*n* = 2); pantothenate kinase-associated neurodegeneration (PKAN) (*n* = 2); bone marrow failure syndrome type 3 (*n* = 1); neuropathy, ataxia, retinitis pigmentosa (NARP) (*n* = 1); Jalili syndrome (*n* = 1), and a presumed mitochondrial DNA depletion syndrome (*n* = 1), as shown in Fig. 2. Regarding Usher syndrome, type II was the most frequent phenotype (48%), followed by type I (32%) and type IV (7%), with 13% of families remaining genetically unsolved.

### Genetic findings

Disease-causing variants were identified in 86/100 families, hereby referred to as the solved cases, for a diagnostic yield of 86.0% (87.1% for Usher and 94.7% for Bardet-Biedl, the most common diagnoses). The most frequently implicated gene in cases of Usher syndrome was *USH2A*, containing disease-causing biallelic variants for 33.9% of families, followed by *MYO7A* in 24.2% of all families. For Bardet-Biedl syndrome, *BBS1* was the most commonly mutated gene (52.6% of families), followed by *BBS10* (21.1%). Further information on the diagnostic yield and all involved genes per diagnosis can be found in Table 2. All solved cases except for the mitochondrial DNA-dependent syndromes were associated with autosomal recessive inheritance. In such cases, a single disease-causing variant in homozygosity was identified in 65% of families (*n* = 54), while 35% (*n* = 29) harbored 2 different variants in compound heterozygosity. Please refer to Supplementary Table 1 for a detailed description.

**Table 2:** Diagnostic yield and causative gene of syndromic RP (data presented *per* family)

Diagnosis	Genetic testing result			Gene	N (%)
	Solved	Unsolved	Total		
Usher	54 (87.1%)	8 (12.9%)	62 (100%)	*ADGRV1*	9 (14.5%)
				*ARSG*	4 (6.5%)
				*CDH23*	3 (4.8%)
				*MYO7A*	15 (24.2%)
				*PCDH15*	1 (1.6%)
				*USH1G*	1 (1.6%)
				*USH2A*	21 (33.9%)
				Unsolved	8 (12.9%)
Bardet-Biedl	18 (94.7%)	1 (5.3%)	19 (100%)	*BBS1*	10 (52.6%)
				*BBS2*	1 (5.3%)
				*BBS10*	4 (21.1%)
				*MKKS*	1 (5.3%)
				*SDCCAG8*	1 (5.3%)
				*TTC8*	1 (5.3%)
				Unsolved	1 (5.3%)
Senior- Løken	5 (71.4%)	2 (28.6%)	7 (100%)	*NPHP1*	2 (28.6%)
				*SDCCAG8*	1 (14.3%)
				*TRAF3IP1*	1 (14.3%)
				*WDR19*	1 (14.3%)
				Unsolved	2 (28.6%)
PKAN	1 (50%)	1 (50%)	2 (100%)	*PANK2*	1 (50%)
				Unsolved	1 (50%)
Kearns-Sayre	2 (100%)	0 (0%)	2 (100%)	mtDNA^a^	2 (100%)
*ARL2BP*-associated ciliopathy	2 (100%)	0 (0%)	2 (100%)	*ARL2BP*	2 (100%)
PHARC	1 (50%)	1 (50%)	2 (100%)	*ABHD12*	1 (50%)
				Unsolved	1 (50%)
Bone marrow failure syndrome 3	1 (100%)	0 (0%)	1 (100%)	*DNAJC21*	1 (100%)
Jalili	1 (100%)	0 (0%)	1 (100%)	*CNNM4*	1 (100%)
NARP	1 (100%)	0 (0%)	1 (100%)	*MT-ATP6*	1 (100%)
MDS	0 (0%)	1 (100%)	1 (100%)	Unsolved	1 (100%)
Total	86 (86%)	14 (14%)	100 (100%)		100 (100%)

*PKAN*, pantothenate kinase-associated neurodegeneration; *NARP*, neuropathy, ataxia, and retinitis pigmentosa; *PHARC*, polyneuropathy, hearing loss, ataxia, retinitis pigmentosa, and cataract; *MDS*, mitochondrial DNA depletion syndrome

^a^Large deletion of mitochondrial DNA involving several genes

A total of 81 unique variants were identified in 25 different genes, 22 of which are novel and herein reported for the first time. The pathogenic variant c.920_923dup p.(His308Glnfs*16) was the most frequently encountered variant in *USH2A-associated* Usher syndrome (*n* = 5/5; families/patients), while c.397dup p.(His133Profs*7) was the most frequent variant for *MYO7A*-associated cases (*n* = 4/7; families/patients). For Bardet-Biedl syndrome, the *BBS1* pathogenic variant c.1169 T > G p.(Met390Arg) was the most commonly identified causative variant (*n* = 9/10; families/patients). A detailed description of all identified genetic variants is available in Table 3.

**Table 3:** Genetic data of identified variants

dbSNP	Nucleotide change	Protein change	Variant type	Predicted effect	ACMG classification (applied criteria^A^)	Count in cohort (*n* of patients/families)	First report
***ABHD12*** (NM_001042472.3)							
	c.728G > A	p.(Trp243*)	SNV	Nonsense	Likely pathogenic (PVS1, PM2)	1 / 1	This study
***ADGRV1*** (NM_032119.4)							
	c.(17019 + 1_17020-1)_(17856 + 1_17857-1)dup		CNV	Exon 79–83 duplication	Likely pathogenic	3 / 2	This study
rs757696771	c.17668_17669del	p.(Met5890Valfs*10)	Indel	Frameshift	Pathogenic (PVS1, PS4, PM2, PP5)	3 / 3	PMID: 21569298
rs746618021	c.2864C > A	p.(Ser955*)	SNV	Nonsense	Pathogenic (PVS1, PS4, PM2, PP5)	1 / 1	PMID: 22147658
rs397517429	c.2870dup	p.(Asn957Lysfs*10)	Indel	Frameshift	Pathogenic (PVS1, PM2, PP5)	1 / 1	This study
	c.6515C > G	p.(Ser2172*)	SNV	Nonsense	Likely pathogenic (PVS1, PM2)	1 / 1	This study
	c.7336del	p.(Glu2446Asnfs*21)	Indel	Frameshift	Likely pathogenic (PVS1, PM2)	1 / 1	This study
	c.9484G > T	p.(Glu3162*)	SNV	Nonsense	Likely pathogenic (PVS1, PM2)	2 / 1	This study
	c.17669del	p.(Met5890Valfs*10)	Indel	Frameshift	Likely pathogenic (PVS1, PM2)	5 / 4	This study
	c.8832del	p.(Gly2945Valfs*2)	Indel	Frameshift	Likely pathogenic (PVS1, PM2)	1 / 1	This study
***ARL2BP*** (NM_012106.4)							
rs199830550	c.207 + 1G > A	p.?	SNV	Splicing	Pathogenic (PVS1, PM2, PM3, PP5)	2 / 2	PMID: 28041643
***ARSG*** (NM_001267727.2)							
rs751461705	c.1326del	p.(Ser443Alafs*12)	Indel	Frameshift	Pathogenic (PVS1, PS3, PM2, PM3, PP5)	4 / 3	PMID: 33300174
rs141748845	c.253 T > C	p.(Ser85Pro)	SNV	Missense	Pathogenic (PM2, PM3, PP3, PP5)	1 / 1	PMID: 33300174
rs1244718647	c.338G > A	p.(Gly113Asp)	SNV	Missense	Pathogenic (PM2, PM3 PP3, PP5)	1 / 1	PMID: 33300174
***BBS1*** (NM_024649.5)							
rs113624356	c.1169 T > G	p.(Met390Arg)	SNV	Missense	Pathogenic (PS3, PM2, PM3, PP1, PP3, PP5)	10 / 9	PMID: 12118255
rs1014835928	c.1318C > T	p.(Arg440*)	SNV	Nonsense	Pathogenic (PVS1, PM2, PM3, PP5)	1 / 1	PMID: 12677556
	c.863 T > G	p.(Leu288Arg)	SNV	Missense	Likely pathogenic (PM2, PM3, PP3, PP4)	1 / 1	This study
rs1490351829	c.118del	p.(Cys40Alafs*2)	Indel	Frameshift	Pathogenic (PVS1, PM2, PM3, PP5)	1 / 1	PMID: 27032803
rs121917777	c.1645G > T	p.(Glu549*)	SNV	Nonsense	Pathogenic (PVS1, PM2, PM3, PP5)	3 / 2	PMID: 12118255
	c.17C > G	p.(Ser6*)	SNV	Nonsense	Likely pathogenic (PVS1, PM2)	1 / 1	This study
***BBS2*** (NM_031885.5)							
rs1368647604	c.402del	p.(Ala136Argfs*65)	Indel	Frameshift	Pathogenic (PVS1, PM2, PM3, PP5)	2 / 1	PMID: 15770229
rs121908178	c.943C > T	p.(Arg315Trp)	SNV	Missense	Likely pathogenic (PS3, PM1, PM2, PM3, PM5, PP3, PP5)	2 / 1	PMID: 11567139
***BBS10*** (NM_024685.4)							
rs1057517031	c.1542del	p.(Asp515Ilefs*9)	Indel	Frameshift	Pathogenic (PVS1, PM2, PP5)	3 / 1	PMID: 16582908
rs549625604	c.271dup	p.(Cys91Leufs*15)	Indel	Frameshift	Pathogenic (PVS1, PM2, PM3, PP5)	1 / 1	PMID: 10874630
rs148374859	c.273C > G	p.(Cys91Trp)	SNV?	Missense	Pathogenic (PS3, PM2, PM3, PP5)	2 / 2	PMID: 16582908
	c.1677del	p.(Tyr559*)	Indel	Frameshift	Pathogenic (PVS1, PM2, PM3, PP5)	1 / 1	PMID: 16582908
***CDH23*** (NM_022124.6)							
rs1385831846	c.3579 + 2 T > C	p.?	SNV	Splicing	Pathogenic (PVS1, PS4, PM2, PP5)	1 / 1	PMID: 11138009
rs1306728898	c.6319C > T	p.(Arg2107*)	SNV	Nonsense	Pathogenic (PVS1, PM2, PP5)	1 / 1	PMID: 11090341
rs111033247	c.6049 + 1G > A	p.?	SNV	Splicing	Pathogenic (PVS1, PS4, PM2, PP5)	1 / 1	PMID: 8894709
	c.753 + 2 T > A	p.?	SNV	Splicing	Likely Pathogenic (PVS1, PM2)	1 / 1	This study
***CNNM4*** (NM_020184.4)							
rs74552543	c.971 T > C	p.(Leu324Pro)	SNV	Missense	Pathogenic (PM2, PM3, PP3, PP5)	1 / 1	PMID: 19200527
***DNAJC21*** (NM_001012339.3)							
	c.805C > T	p.(Gln269*)	SNV	Nonsense	Likely pathogenic (PVS1, PM2)	1 / 1	This study
***MKKS*** (NM_170784.3)							
rs768929313	c.748G > A	p.(Gly250Arg)	SNV	Missense	Pathogenic (PM2, PM3, PM5, PP3, PP5)	3 / 1	PMID: 20142850
***MT-ATP6***							
rs199476133	m.8993 T > G	p.(Leu156Arg)	SNV	Missense	Pathogenic (PS2, PM3, PM5, PP3, PP5)	2 / 1	PMID: 2137962
***MYO7A*** (NM_000260.4)							
	c.1529 T > C	p.(Ile510Thr)	SNV	Missense	Likely pathogenic (PM1, PM2, PM3, PP3)	1 / 1	This study
rs111033214	c.3508G > A	p.(Glu1170Lys)	SNV	Missense	Pathogenic (PS4, PM2 PM5, PP3, PP5)	5 / 3	PMID: 10425080
rs111033187	c.397dup	p.(His133Profs*7)	Indel	Frameshift	Pathogenic (PVS1, PM2, PM3, PP5)	7 / 4	PMID: 21569298
rs751769391	c.4489G > C	p.(Gly1497Arg)	SNV	Missense	Pathogenic (PM2, PM5, PP3, PP5)	3 / 3	PMID: 27460420
	c.5510 T > A	p.(Leu1837His)	SNV	Missense	Pathogenic (PM2, PM5, PP3, PP5)	4 / 4	PMID: 36909829
	c.5743-15_5746del	p.(Ala1915fs)	Indel	Frameshift	Likely pathogenic (PVS1, PM2)	1 / 1	This study
rs1591514873	c.6439-1G > A	p.?	SNV	Splicing	Pathogenic (PVS1, PM2, PP5)	3 / 2	PMID: 16199547
rs111033285	c.999 T > G	p.(Tyr333*)	SNV	Nonsense	Pathogenic (PVS1, PS4, PM2, PP5)	1 / 1	PMID: 8900236
	c.1929dup	p.(Pro644Alafs*67)	Indel	Frameshift	Pathogenic (PVS1, PM2, PP5)	2 / 1	PMID: 36909829
rs1173853484	c.6026C > A	p.(Ala2009Asp)	SNV	Missense	Likely pathogenic (PP3, PP5, PM1, PM2, PM5)	1 / 1	PMID: 27460420
***NPHP1*** (NM_001128178.3)							
	c.2065_2074del	p.(Thr689Leufs*37)	Indel	Frameshift	Likely pathogenic (PVS1, PM2)	2 / 2	This study
***NPHP4*** (NM_015102.5)							
rs370946873	c.2956G > A	p.(Gly986Arg)	SNV	Missense	VUS (PM2, PP3)	1 / 1	PMID: 36909829
***NRL*** (NM_001354768.3)							
rs774348345	c.74G > A	p.(Arg25Gln)	SNV	Missense	VUS (PM2, PP2)	1 / 1	This study
***PANK2*** (NM_001386393.1)							
rs779815683	c.1268G > T	p.(Cys423Phe)	SNV	Missense	VUS (PM1, PM2, PP2, PP3)	1 / 1	This study
rs137852959	c.1561G > A	p.(Gly521Arg)	SNV	Missense	Pathogenic (PS3, PM2, PM3, PP1, PP2, PP3, PP5)	1 / 1	PMID: 11479594
rs754521581	c.1070G > C	p.(Arg357Pro)	SNV	Missense	Likely pathogenic (PM1, PM2, PM3, PM5, PP2, PP5)	1 / 1	PMID: 28680084
***PCDH15*** (NM_001384140.1)							
	c.(2220 + 1_2221-1)_(3122 + 1_3123-1)dup		CNV	Exon 19–23 duplication	Likely pathogenic	1 / 1	PMID: 20538994
***SDCCAG8*** (NM_006642.5)							
rs768207230	c.397G > T	p.(Glu133*)	SNV	Nonsense	Pathogenic (PVS1, PM2, PM3, PP5)	2 / 2	This study
***SLC7A14*** (NM_020949.3)							
rs116040996	c.821C > T	p.(Thr274IIe)	SNV	Missense	VUS (PP3, PM2)	1 / 1	This study
***TRAF3IP1*** (NM_015650.4)							
rs778376663	c.916-4A > G	p.?	SNV	Splicing	Likely pathogenic (PP3, PM2, PM3)	2 / 1	PMID: 36909829
***TTC8*** (NM_001288781.1)							
	c.647G > A	p.(Trp216*)	SNV	Missense	Likely pathogenic (PVS1, PM2, PP5)	1 / 1	This study
	c.(?_681-1)_(879 + 1_?)del		CNV	Exon 9–10 deletion	Likely pathogenic	1 / 1	
***USH1G*** (NM_173477.5)							
	c.183 T > A	p.(Cys61*)	SNV	Nonsense	Likely pathogenic (PVS1, PM2)	1 / 1	This study
***USH2A*** (NM_206933.4)							
rs750228923	c.1214del	p.(Asn405Ilefs*3)	Indel	Frameshift	Pathogenic (PVS1, PM2, PM3, PP5)	1 / 1	PMID:16098008
	c.12294 + 1559_14133 + 8144del		CNV	Exon 63–64 deletion	Likely pathogenic	1 / 1	PMID: 28041643
rs998302546	c.14134-3169A > G	p.?	SNV	Splicing	Likely pathogenic (PM2, PM3, PP5)	2 / 1	PMID: 29196752
	c.14423G > A	p.(Cys4808Tyr)	SNV	Missense	VUS (PM1, PM2, PM3)	1 / 1	PMID: 36909829
	c.1879C > T	p.(Gln627*)	SNV	Nonsense	Likely pathogenic (PVS1, PM2)	1 / 1	This study
rs111033334	c.2209C > T	p.(Arg737*)	SNV	Nonsense	Pathogenic (PVS1, PM2, PM3, PP5)	1 / 1	PMID: 17296898
rs80338902	c.2276G > T	p.(Cys759Phe)	SNV	Missense	Pathogenic (PS4, PM1, PM2, PM3, PP1, PP3, PP4, PP5)	1 / 1	PMID: 1968399
	c.(7300 + 1_7301-1)_(9371 + 1_9372-1)del		CNV	Exon 38–47 deletion	Likely pathogenic	3 / 2	
rs202175091	c.10712C > T	p.(Thr3571Met)	SNV	Missense	Pathogenic (PM1, PM2, PM3, PM5, PP1, PP5)	1 / 1	PMID: 17085681
rs527236139	c.11156G > A	p.(Arg3719His)	SNV	Missense	Pathogenic (PP1, PP5, PM2, PM3)	2 / 2	PMID: 20507924
rs397517994	c.14911C > T	p.(Arg4971*)	SNV	Nonsense	Pathogenic (PVS1, PP5, PM2, PM3)	2 / 1	PMID: 10729113
rs758660532	c.15089C > A	p.(Ser5030*)	SNV	Nonsense	Pathogenic (PVS1, PP5, PM2, PM3)	2 / 1	PMID: 10729113
rs80338903	c.2299del	p.(Glu767Serfs*21)	Indel	Frameshift	Pathogenic (PVS1, PP1, PP5, PM2, PM3)	2 / 2	PMID: 9624053
rs1052375050	c.2302 T > C	p.(Cys768Arg)	SNV	Missense	Likely pathogenic (PP3, PM2)	1 / 1	PMID: 36909829
rs759433119	c.2809 + 1G > A	p.?	SNV	Splicing	Pathogenic (PVS1, PP5, PM2, PM3)	2 / 1	PMID: 10729113
rs754374132	c.5278del	p.(Asp1760Metfs*10)	Indel	Frameshift	Pathogenic (PVS1, PP5, PM2, PM3)	1 / 1	PMID: 10729113
rs1571783742	c.7932G > A	p.(Trp2644*)	SNV	Nonsense	Pathogenic (PVS1, PP5, PM2, PM3)	2 / 2	PMID: 10729113
rs748465849	c.907C > A	p.(Arg303Ser)	SNV	Missense	Pathogenic (PP5, PM2, PM3, PM5)	3 / 3	PMID: 14970843
rs397518043	c.920_923dup	p.(His308Glnfs*16)	Indel	Frameshift	Pathogenic (PVS1, PP5, PM2, PM3)	5 / 5	PMID: 18641288
rs111033263	c.9799 T > C	p.(Cys3267Arg)	SNV	Missense	Likely pathogenic (PP3, PP5,PM2, PM3, PM5)	1 / 1	PMID: 17085681
	c.9315del	p.(Val3106Trpfs*54)	Indel	Frameshift	Likely pathogenic (PVS1, PM2)	1 / 1	PMID: 36909829
rs150982499	c.5039A > G	p.(Lys1680Arg)	SNV	Missense	VUS (PM1, PM2)	1 / 1	PMID: 28912962
***WDR19*** (NM_025132.4)							
rs1020915921	c.2704-2A > C	p.?	SNV	Splicing	Pathogenic (PVS1, PP5, PM2)	1 / 1	PMID: 16199547
rs387906980	c.1649 T > C	p.(Leu550Ser)	SNV	Missense	Pathogenic (PP1, PP3, PP5, PM2, PM3)	1 / 1	PMID: 22019273

^A^Each ACMG pathogenicity criterion is weighted as very strong (PVS), strong (PS), moderate (PM), or supporting (PP)

*dbSNP*, single nucleotide polymorphism database; *PMID*, PubMed identifier; *VUS*, variant of uncertain significance

### Ocular findings

**Fig. 3:**
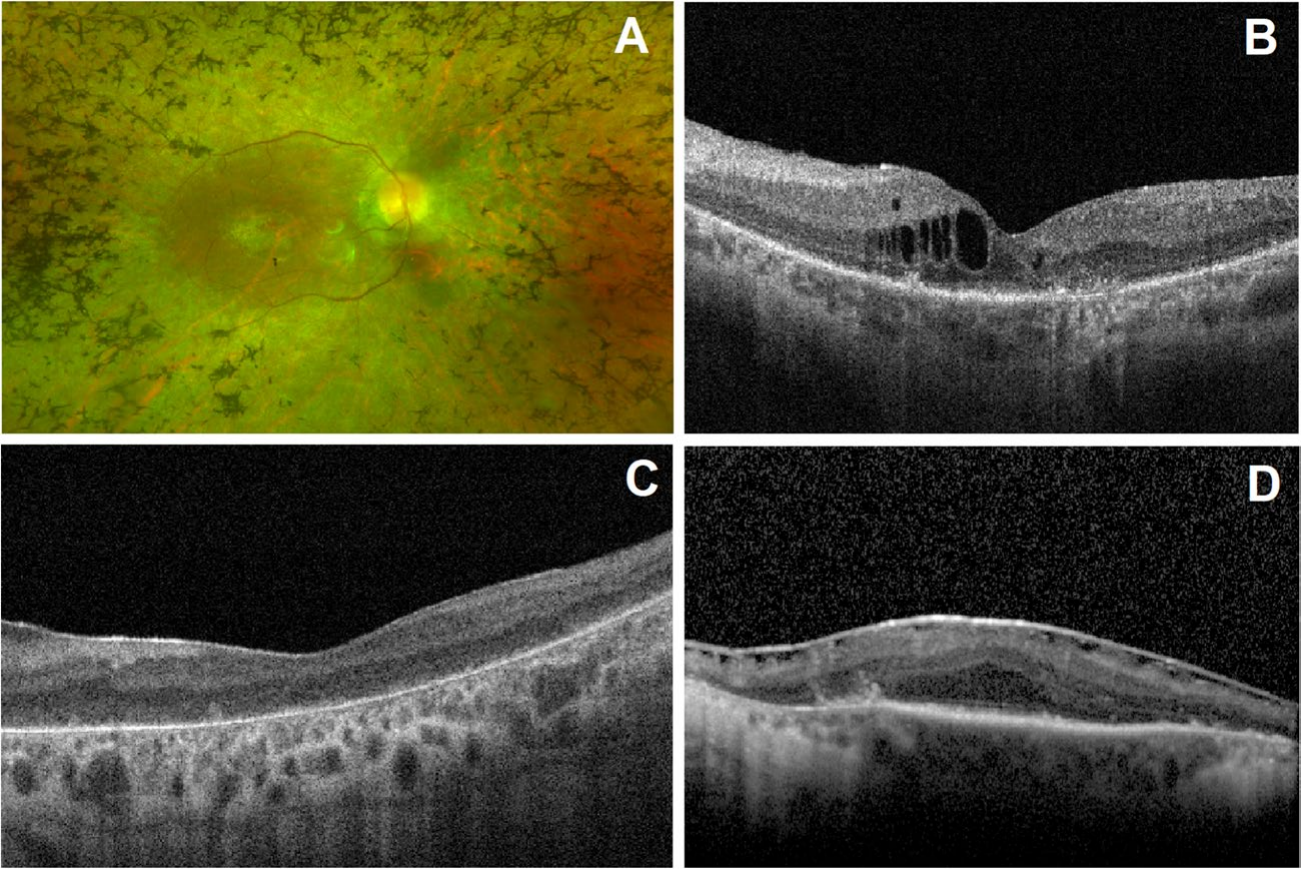
Ultra-widefield color fundus photography and spectral-domain optical coherence tomography (OCT) imaging of syndromic RP patients. (**A**) Classic fundus findings of retinitis pigmentosa: blood vessel attenuation and bone spicule hyperpigmentation in an Usher syndrome patient (macular atrophy is also present). (**B**) Cystoid macular edema present in *USH2A*-associated Usher syndrome. (**C**) OCT imaging displaying foveal atrophy of the outer retinal layers and RPE/Bruch’s membrane complex in *BBS1*-associated Bardet-Biedl syndrome. (**D**) Epiretinal membrane causing loss of foveal depression and presence of ectopic inner foveal layers in an Usher syndrome patient

**Fig. 4:**
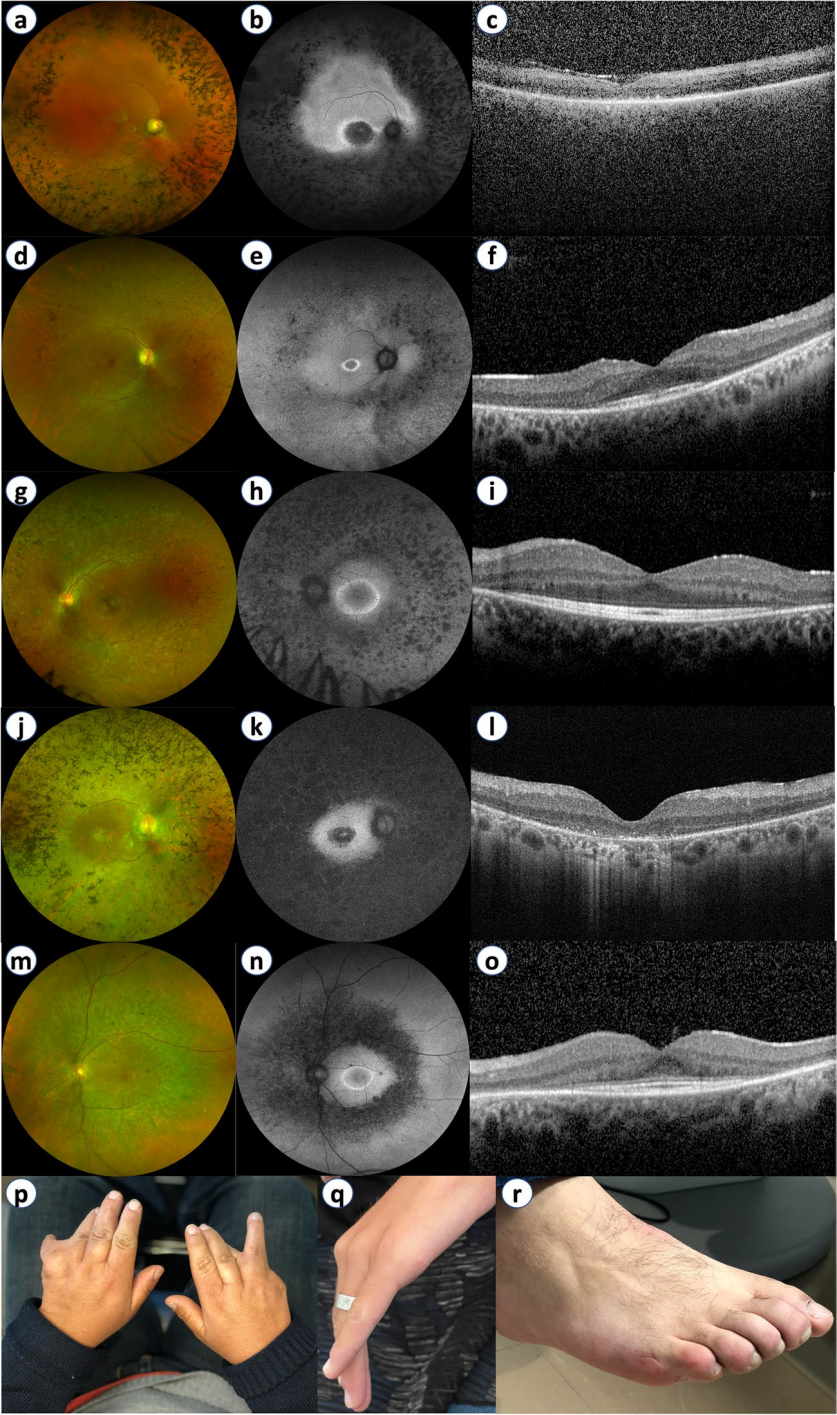
(**a**–**o**) Ultra-widefield color fundus photography (UWF-CFP), ultra-widefield fundus autofluorescence (UWF-FAF), and spectral-domain optical coherence tomography (OCT) imaging of five syndromic RP patients: (**a**–**c**) *BBS10*-associated Bardet-Biedl syndrome; (**d**–**f**) *SDCCAG8-*associated Bardet-Biedl syndrome; (**g**–**i**) *MYO7A*-associated Usher syndrome; (**j–l**) *USH2A*-associated Usher syndrome; (**m**–**o**) *ARSG*-associated Usher syndrome. Bone spicule hyperpigmentation and patches of outer retinal atrophy seen on UWF-CFP (**a**, **d**, **g**, **j**, and **m**) directly correspond to hypoautofluorescent patches on UWF-FAF (**b**, **e**, **h**, **k**, and n). The parafoveal hyperautofluorescent ring (**e**, **h**, and **n**) directly correlates to the extent of outer retinal layer preservation in the corresponding OCT imaging (**f**, **l**, and **o**). Foveal atrophy of the outer retinal layers and RPE/Bruch’s membrane complex are typically found earlier in Bardet-Biedl syndrome (**c**) comparatively to Usher syndrome (**i** and **o**), where it is usually found in the latter stages of the disease. (**p**–**r**) Clinical photographs depicting congenital limb malformations in Bardet-Biedl syndrome: syndactyly in *BBS10*-associated Bardet-Biedl syndrome (**p**); residual hand appendage in *BBS1*-associated Bardet-Biedl syndrome (**q**); and patient with *BBS1*-associated Bardet-Biedl syndrome born with clinically evident polydactyly, subject to correcting surgery during childhood (**r**)

One hundred twenty-two patients were followed for a median period of 43 months. Best-corrected visual acuity (BCVA) records were available at both baseline and follow-up for 99 patients (198 eyes), followed for a median period of 62.0 months. The mean BCVA for this group was at baseline 56.5 Early Treatment Diabetic Retinopathy Study (ETDRS) letters (Snellen equivalent ~ 20/80), declining to 44.9 ETDRS letters (Snellen equivalent ~ 20/125) at the last available follow-up, a statistically significant change (*p* < 0.001). Ocular comorbidities were identified in 39.1% of all eyes, the most frequent being cystoid macular edema, present in 13.6% of eyes, followed by epiretinal membrane (9.9% of eyes) (Fig. 3). Figure 4 depicts the retinal phenotype of 5 patients from our cohort.

## Discussion

Genetic profiling of IRDs is of major importance for patients and, through genetic counseling, for family members as well. Nevertheless, constraints in access to genetic testing may hinder the goal of obtaining a molecular diagnosis for every affected patient [17]. A paradigm shift is in progress, with a recent increase in the number of publications contributing to improve knowledge of the genetic landscape of IRDs in Portugal [13, 18–24]. One of such publications included a cohort of 230 Portuguese families with IRDs, but only 23 probands had syndromic RP [13]. In this nationwide, multicenter study including 122 patients from 100 families, we describe the genetic landscape of syndromic RP in Portugal.

Overall, disease-causing variants were identified in 86/100 families for a diagnostic yield of 86%. Even though this figure is much higher than what is usually obtained for non-syndromic forms of the disease [7, 25], it is in line with a previous study by Karali et al. [10], reporting genetic testing sensitivity upwards of 80% for syndromic IRDs.

Given the geographic proximity between Portugal and Spain, as well as the genetic similarities observed between its inhabitants [26], studies on the genetic landscape of syndromic RP in Spanish cohorts are a natural reference for comparison purposes, and thus, one could anticipate somewhat similar genetic findings for a Portuguese cohort. As expected, Usher (*n* = 62 families) and Bardet-Biedl (*n* = 19 families) syndromes were found to be the most frequent causes of syndromic RP in our cohort. *USH2A* and *MYO7A* variants were the major causes of Usher syndrome type II and type I, respectively. Similar findings were reported by Perea-Romero et al. [7] in their large Spanish cohort (*n* = 577 syndromic IRD families) and are observed as well in most studies from different populations [27–29]. Additionally, the *BBS1* variant c.1169 T > G p.(Met380Arg) was the most frequently identified causative variant for Bardet-Biedl cases. This is in line with other Caucasian cohorts, where it was shown that ~ 80% of patients with *BBS1*-related disease carry this pathogenic variant [30, 31].

Even so, significant differences were found in the genetic architecture of Usher syndrome for the present cohort, as illustrated by the comparatively high prevalence of *ADGRV1* variants, present in 14.5% of families, but found to be less common in Spanish [8] or North American [25] cohorts. Conversely, *PCDH15* mutations were a prevalent cause of type 1 Usher syndrome, responsible for over 15% of such cases in both Spanish [7] and North American [25] cohorts, but were identified in just a single family in this study.

Eighty-one distinct genetic variants in 25 different genes were identified, 22 of which are novel. For *USH2A*-associated Usher syndrome, the most prevalent disease-causing variant was c.920_923dup p.(His308Glnfs*16), previously reported in multiple European cohorts [32–34]. The frameshift variant c.397dup p.(His133Profs*7), first reported by Bonnet et al. [35], was the most prevalent cause of *MYO7A*-associated Usher syndrome. The *ADGRV1* gene contained the most novel variants (*n* = 7), all of which were disease-causing, i.e., ACMG class IV or V. The remaining novel variants were distributed across 13 different genes (Table 3).

We found that most patients (61.5%) experience a symptomatic onset of vision loss during the first 20 years of age, with Bardet-Biedl syndrome patients reporting the earliest visual symptom onset, i.e., within the first decade of life (Table 1). Although a direct comparison cannot be established, this appears to be before than most cases of non-syndromic RP, where a mean age of onset of 19.5 ± 12.6 years and 23.2 ± 16.6 years has been reported by Colombo et al. for autosomal dominant and autosomal recessive non-syndromic RP, respectively, in a large Italian cohort [36]. A mean loss of 11.6 ETDRS letters (*p* < 0.001) was observed over a follow-up period of 62.0 months, corresponding to an annual reduction in BCVA of 2.24 letters. A similar reduction (2.3 letters) was previously reported by Iftikhar et al. [37] in their cohort of non-syndromic RP patients, illustrating the slowly progressive nature of the disease. Cystoid macular edema was present in 13.6% of eyes. The previously reported prevalence for this comorbidity is widely variable, ranging from ~ 5% [38] to 50.9% [39] of eyes (in non-syndromic RP), and has been noticed not to differ significantly between syndromic or non-syndromic RP [20]. Regardless, ophthalmologists should be aware of the importance of screening patients for the presence of this potentially treatable condition [20, 39].

Our study presents some limitations. First, the absence of standardization in multimodal retinal imaging across different contributing HCPs may have led to differences in the reporting of comorbidities such as cystoid macular edema and epiretinal membrane, as patients were not required to have performed regular optical coherence tomography (OCT) imaging to be included in the cohort. Also, not all Portuguese regions were represented in this cohort, as there were 4 districts for which no patients were included (Fig. 1). Naturally, there is a selection bias toward patients who can visit the ophthalmology clinics of the contributing HCPs. Patients with severe comorbidities and those living in more remote areas may have difficulties accessing these specialized centers and may be underrepresented in this sample. Nevertheless, we were able to enroll a large number of syndromic RP patients from six different HCPs, providing genetic data from 100 families.

In conclusion, as ophthalmology takes a deep dive into precision medicine, nationwide efforts to improve knowledge of the genetic background of IRDs are of utmost importance. The present study illustrates the diverse genetic landscape and provides reference data for syndromic RP in Portugal. Twenty-two novel variants in syndromic RP-associated genes are herein reported for the first time, thus contributing to expand the mutational spectrum of syndromic RP.

### Supplementary information

Below is the link to the electronic supplementary material.

Supplementary file1 (DOCX 35 KB)
