# Supplementary material for: Genetic profile of syndromic retinitis pigmentosa in Portugal
Source: Graefes Arch Clin Exp Ophthalmol. 2024 Jan 8;262(6):1883–97. doi: 10.1007/s00417-023-06360-2 (PMC11106148; doi:10.1007/s00417-023-06360-2)
Supplement: Supplementary file 1 — Supplementary file1 (DOCX 35 KB) [file 417_2023_6360_MOESM1_ESM.docx]

**Supplemental Table 1.** Genetic data of genetically solved families

|  | Number of affected individuals | Zygosity | Gene | Variant #1 | Variant #2 |
| --- | --- | --- | --- | --- | --- |
| Usher | | | | | |
| Family #2 | 1 | HMZ | *USH2A* | c.10712C>T p.(Thr3571Met) | c.10712C>T p.(Thr3571Met) |
| Family #3 | 1 | HMZ | *CDH23* | c.3579+2T>C p.? | c.3579+2T>C p.? |
| Family #9 | 1 | HMZ | *USH2A* | c.7932G>A p.(Trp2644*) | c.7932G>A p.(Trp2644*) |
| Family #10 | 2 | HMZ | *USH2A* | c.(7300+1_7301-1)_(9371+1_9372-1)del | c.(7300+1_7301-1)_(9371+1_9372-1)del |
| Family #12 | 1 | HMZ | *USH1G* | c.183T>A p.(Cys61*) | c.183T>A p.(Cys61*) |
| Family #13 | 2 | HMZ | *USH2A* | c.14911C>T p.(Arg4971*) | c.14911C>T p.(Arg4971*) |
| Family #16 | 1 | HMZ | *MYO7A* | c.397dup p.(His133Profs*7) | c.397dup p.(His133Profs*7) |
| Family #20 | 1 | HMZ | *ADGRV1* | c.7336del p.(Glu2446Asnfs*21) | c.7336del p.(Glu2446Asnfs*21) |
| Family #25 | 2 | HMZ | *ARSG* | c.1326del p.(Ser443Alafs*12) | c.1326del p.(Ser443Alafs*12) |
| Family #26 | 1 | HMZ | *ADGRV1* | c.17668_17669del p.(Met5890Valfs*10) | c.17668_17669del p.(Met5890Valfs*10) |
| Family #27 | 1 | C.HTZ | *USH2A* | c.907C>A p.(Arg303Ser) | c.1879C>T p.(Gln627*) |
| Family #28 | 1 | C.HTZ | *ADGRV1* | c.6515C>G p.(Ser2172*) | c.(17019+1_17020-1)_(17856+1_17857-1)dup |
| Family #33 | 1 | HMZ | *MYO7A* | c.6439-1G>A p.? | c.6439-1G>A p.? |
| Family #34 | 1 | C.HTZ | *USH2A* | c.5278del p.(Asp1760Metfs*10) | c.11156G>A p.(Arg3719His) |
| Family #37 | 2 | HMZ | *USH2A* | c.2809+1G>A (IVS13+1G>A) p.? | c.2809+1G>A (IVS13+1G>A) p.? |
| Family #39 | 1 | HMZ | *ARSG* | c.1326del p.(Ser443Alafs*12) | c.1326del p.(Ser443Alafs*12) |
| Family #42 | 1 | HMZ | *MYO7A* | c.5510T>A p.(Leu1837His) | c.5510T>A p.(Leu1837His) |
| Family #45 | 1 | HMZ | *MYO7A* | c.999T>G p.(Tyr333Ter) | c.999T>G p.(Tyr333Ter) |
| Family #46 | 1 | HMZ | *ADGRV1* | c.17668_17669del p.(Met5890Valfs*10) | c.17668_17669del p.(Met5890Valfs*10) |
| Family #40 | 1 | C.HTZ | *MYO7A* | c.1529T>C p.(Ile510Thr) | c.4489G>C p.(Gly1497Arg) |
| Family #41 | 1 | HMZ | *USH2A* | c.920_923dup p.(His308Glnfs*16) | c.920_923dup p.(His308Glnfs*16) |
| Family #49 | 1 | C.HTZ | *USH2A* | c.907C>A p.(Arg303Ser) | c.2209C>T p.(Arg737*) |
| Family #54 | 1 | HMZ | *USH2A* | c.920_923dup p.(His308Glnfs*16) | c.920_923dup p.(His308Glnfs*16) |
| Family #56 | 1 | HMZ | *MYO7A* | c.4489G>C p.(Gly1497Arg) | c.4489G>C p.(Gly1497Arg) |
| Family #58 | 1 | C.HTZ | *CDH23* | c.6319C>T p.(Arg2107*) | c.6049+1G>A p.? |
| Family #61 | 1 | HMZ | *MYO7A* | c.3508G>A p.(Glu1170Lys) | c.3508G>A p.(Glu1170Lys) |
| Family #62 | 1 | HMZ | *USH2A* | c.(7300+1_7301-1)_(9371+1_9372-1)del | c.(7300+1_7301-1)_(9371+1_9372-1)del |
| Family #63 | 1 | HMZ | *MYO7A* | c.5743-15_5746del p.(Ala1915fs) | c.5743-15_5746del p.(Ala1915fs) |
| Family #68 | 1 | C.HTZ | *MYO7A* | c.5510T>A p.(Leu1837His) | c.6026C>A p.(Ala2009Asp) |
| Family #69 | 1 | HMZ | *CDH23* | c.753+2T>A p.? | c.753+2T>A p.? |
| Family #70 | 1 | HMZ | *ADGRV1* | c.17668_17669del p.(Met5890Valfs*10) | c.17668_17669del p.(Met5890Valfs*10) |
| Family #71 | 1 | HMZ | *USH2A* | c.920_923dup p.(His308Glnfs*16) | c.920_923dup p.(His308Glnfs*16) |
| Family #64 | 1 | C.HTZ | *USH2A* | c.920_923dup p.(His308Glnfs*16) | c.1214del p.(Asn405Ilefs*3) |
| Family #74 | 2 | HMZ | *MYO7A* | c.3508G>A p.(Glu1170Lys) | c.3508G>A p.(Glu1170Lys) |
| Family #77 | 1 | C.HTZ | *USH2A* | c.11156G>A p.(Arg3719His) | c.2276G>T p.(Cys759Phe) |
| Family #78 | 2 | C.HTZ | *MYO7A* | c.6439-1G>A p.? | c.3508G>A p.(Glu1170Lys) |
| Family #79 | 1 | HMZ | *USH2A* | c.2299delG p.(Glu767Serfs*21) | c.2299delG p.(Glu767Serfs*21) |
| Family #80 | 2 | HMZ | *ADGRV1* | c.(17019+1_17020-1)_(17856+1_17857-1)dup | c.(17019+1_17020-1)_(17856+1_17857-1)dup |
| Family #81 | 1 | HMZ | *USH2A* | c.9799T>C p.(Cys3267Arg) | c.9799T>C p.(Cys3267Arg) |
| Family #82 | 2 | C.HTZ | *ADGRV1* | c.9484G>T p.(Glu3162*) | c.17669del p.(Met5890Valfs*10) |
| Family #83 | 1 | C.HTZ | *ADGRV1* | c.2870dup p.(Asn957Lysfs*10) | c.8832del p.(Gly2945Valfs*2) |
| Family #84 | 1 | HMZ | *USH2A* | c.920_923dup p.(His308Glnfs*16) | c.920_923dup p.(His308Glnfs*16) |
| Family #86 | 1 | C.HTZ | *MYO7A* | c.4489G>C p.(Gly1497Arg) | c.5510T>A p.(Leu1837His) |
| Family #87 | 3 | HMZ | *MYO7A* | c.397dup p.(His133Profs*7) | c.397dup p.(His133Profs*7) |
| Family #89 | 1 | HMZ | *ARSG* | c.1326del p.(Ser443Alafs*12) | c.1326del p.(Ser443Alafs*12) |
| Family #90 | 2 | C.HTZ | *USH2A* | c.15089C>A p.(Ser5030Ter) | c.14134-3169A>G p.? |
| Family #93 | 1 | C.HTZ | *ARSG* | c.253T>C p.(Ser85Pro) | c.338G>A p.(Gly113Asp) |
| Family #96 | 1 | HMZ | *ADGRV1* | c.2864C>A p.(Ser955Ter) | c.2864C>A p.(Ser955Ter) |
| Family #97 | 1 | C.HTZ | *MYO7A* | c.5510T>A p.(Leu1837His) | c.397dup p.(His133Profs*7) |
| Family #98 | 1 | C.HTZ | *USH2A* | c.2302T>C p.(Cys768Arg) | c.12294+1559_14133+8144del p.? |
| Family #100 | 2 | C.HTZ | *MYO7A* | c.397dup p.(His133Profs*7) | c.1929dup p.(Pro644Alafs*67) |
| Family #65 | 1 | HMZ | *PCDH15* | c.(2220+1_2221-1)_(3122+1_3123-1)dup | c.(2220+1_2221-1)_(3122+1_3123-1)dup |
| Family #66 | 1 | HMZ | *USH2A* | c.907C>A p.(Arg303Ser) | c.907C>A p.(Arg303Ser) |
| Family #72 | 1 | C.HTZ | *USH2A* | c.7932G>A p.(Trp2644Ter) | c.9315del p.(Val3106Trpfs*54) |
| Bardet-Biedl | | | | | |
| Family #6 | 1 | C.HTZ | *BBS1* | c.863T>G p.(Leu288Arg) | c.1169T>G p.(Met390Arg) |
| Family #17 | 1 | HMZ | *BBS10* | c.273C>G p.(Cys91Trp) | c.273C>G p.(Cys91Trp) |
| Family #18 | 1 | HMZ | *BBS1* | c.1169T>G p.(Met390Arg) | c.1169T>G p.(Met390Arg) |
| Family #19 | 1 | C.HTZ | *BBS1* | c.1169T>G p.(Met390Arg) | c.17C>G p.(Ser6*) |
| Family #21 | 1 | C.HTZ | *BBS10* | c.273C>G p.(Cys91Trp) | c.1677del p.(Tyr559Ter) |
| Family #23 | 3 | HMZ | *BBS10* | c.1542del p.(Asp515Ilefs*9) | c.1542del p.(Asp515Ilefs*9) |
| Family #31 | 1 | C.HTZ | *BBS1* | c.1318C>T p.(Arg440*) | c.1645G>T p.(Glu549*) |
| Family #32 | 2 | C.HTZ | *BBS1* | c.1169T>G p.(Met390Arg) | c.1645G>T p.(Glu549*) |
| Family #35 | 1 | HMZ | *BBS10* | c.271dup p.(Cys91Leufs15) | c.271dup p.(Cys91Leufs15) |
| Family #38 | 1 | HMZ | *BBS1* | c.1169T>G p.(Met390Arg) | c.1169T>G p.(Met390Arg) |
| Family #47 | 3 | HMZ | *MKKS* | c.748G>A p.(Gly250Arg) | c.748G>A p.(Gly250Arg) |
| Family #50 | 1 | C.HTZ | *TTC8* | c.647G>A p.(Trp216) | c.(?_681-1)_(879 1_?)del |
| Family #55 | 1 | HMZ | *SDCCAG8* | c.397G>T p.(Glu133*) | c.397G>T p.(Glu133*) |
| Family #73 | 1 | C.HTZ | *BBS1* | c.1169T>G p.(Met390Arg) | c.118del p.(Cys40Alafs*2) |
| Family #75 | 2 | C.HTZ | *BBS2* | c.402del p.(Ala136Argfs*65) | c.943C>T p.(Arg315Trp) |
| Family #85 | 1 | HMZ | *BBS1* | c.1169T>G p.(Met390Arg) | c.1169T>G p.(Met390Arg) |
| Family #94 | 1 | HMZ | *BBS1* | c.1169T>G p.(Met390Arg) | c.1169T>G p.(Met390Arg) |
| Family #99 | 1 | HMZ | *BBS1* | c.1169T>G p.(Met390Arg) | c.1169T>G p.(Met390Arg) |
| Senior-Løken | | | | | |
| Family #1 | 1 | HMZ | *SDCCAG8* | c.397G>T p.(Glu133*) | c.397G>T p.(Glu133*) |
| Family #29 | 1 | HMZ | *NPHP1* | c.2065_2074del p.(Thr689Leufs*37) | c.2065_2074del p.(Thr689Leufs*37) |
| Family #67 | 1 | HMZ | *NPHP1* | c.2065_2074del p.(Thr689Leufs*37) | c.2065_2074del p.(Thr689Leufs*37) |
| Family #91 | 2 | HMZ | *TRAF3IP1* | c.916-4A>G p.? | c.916-4A>G p.? |
| Family #92 | 1 | C.HTZ | *WDR19* | c.2704-2A>C p.? | c.1649T>C p.(Leu550Ser) |
| PKAN | | | | | |
| Family #48 | 1 | C.HTZ | *PANK2* | c.1070G>C p.(Arg357Pro) | c.1561G>A p.(Gly521Arg) |
| *ARL2BP-*associated ciliopathy | | | | | |
| Family #76 | 1 | HMZ | *ARL2BP* | c.207+1G>A p.? | c.207+1G>A p.? |
| Family #88 | 1 | HMZ | *ARL2BP* | c.207+1G>A p.? | c.207+1G>A p.? |
| PHARC | | | | | |
| Family #44 | 1 | HMZ | *ABHD12* | c.728G>A p.(Trp243*) | c.728G>A p.(Trp243*) |
| Bone Marrow Failure Syndrome 3 | | | | | |
| Family #5 | 1 | HMZ | *DNAJC21* | c.805C>T p.(Gln269*) | c.805C>T p.(Gln269*) |
| Jalili | | | | | |
| Family #4 | 1 | HMZ | *CNNM4* | c.971T>C p.(Leu324Pro) | c.971T>C p.(Leu324Pro) |
| NARP | | | | | |
| Family #95 | 2 | HMZ | *MT-ATP6* | m.8993 T>G | Non-applicable  (Mitochondrial DNA) |

PKAN: pantothenate kinase-associated neurodegeneration; NARP: Neuropathy, ataxia, and retinitis pigmentosa; PHARC: polyneuropathy, hearing loss, ataxia, retinitis pigmentosa and cataract;HMZ – Homozygous; C.HTZ – Compound Heterozygous. Does not include variant data regarding the two Kearns-Sayre families (both harbored large mitochondrial DNA deletions across several genes)
